# A Fetal and an Intra-Amniotic Inflammatory Response Is More Severe in Preterm Labor than in Preterm PROM in the Context of Funisitis: Unexpected Observation in Human Gestations

**DOI:** 10.1371/journal.pone.0062521

**Published:** 2013-05-01

**Authors:** Chan-Wook Park, Bo Hyun Yoon, Joong Shin Park, Jong Kwan Jun

**Affiliations:** Department of Obstetrics and Gynecology, Seoul National University College of Medicine, Seoul, Korea; Université de Montréal, Canada

## Abstract

**Objective:**

Although intra-amniotic(IA) infection is present in both preterm labor and intact membranes(PTL) and preterm premature rupture of membranes(preterm-PROM), it is more common in preterm-PROM than in PTL. Microorganisms and their products in the amniotic-cavity can elicit an inflammatory-response in fetus as well as in amniotic-cavity in the progression of acute histologic chorioamnionitis(acute-HCA). A fundamental question is whether a fetal and an IA inflammatory-response is more severe in preterm-PROM than in PTL, in the same-context of acute-HCA with or without fetal-involvement. The purpose of current-study was to answer this-question.

**Study-Design:**

Study population consisted of 213 singleton preterm-gestations(<34 weeks) delivered within 4 days of amniocentesis due to PTL(120 cases) or preterm-PROM(93 cases). The intensity of fetal and IA inflammatory-responses was compared between PTL and preterm-PROM, according to placental inflammatory conditions:1)placenta without inflammatory-lesion;2)acute-HCA without funisitis;3)acute-HCA with funisitis. IA inflammatory response was assessed by amniotic-fluid(AF) matrix metalloproteinase-8(MMP-8), and fetal inflammatory response(FIR) by umbilical-cord plasma(UCP) C-reactive protein(CRP) at birth.

**Results:**

1) Patients with preterm-PROM had higher rates of IA infection, acute-HCA, and acute-HCA with funisitis than those with PTL did(*p*<.01 for each);2) there were no significant differences in the intensity of fetal and IA inflammatory-responses and the rate of cervical dilatation≥3 cm or 4 cm between patients with PTL and those with preterm-PROM in the context of both placenta without inflammatory-lesion and acute-HCA without funisitis(*p*>.05 for each);3) however, acute-HCA with funisitis was associated with a significantly higher median AF MMP-8 and UCP CRP concentration and higher rate of cervical dilatation≥3 cm or 4 cm in PTL than in preterm-PROM(AF MMP-8, 675 ng/mlvs.417 ng/ml; UCP CRP, 969 ng/mlvs.397 ng/ml;each for *p*<.05), despite less common IA infection in PTL than in preterm-PROM(29%vs.57%;*p*<.05).

**Conclusions:**

A fetal and an IA inflammatory-response is more severe in PTL than in preterm-PROM in the context of funisitis, despite less common IA infection. This unexpected observation may indicate the fundamental difference in the pathogenesis between PTL and preterm-PROM.

## Introduction

Microbial invasion of amniotic cavity (MIAC) can be present in both patients with preterm labor and intact membranes (PTL) and those with preterm premature rupture of membranes (preterm-PROM) [1]–[8]. However, it is more common in preterm-PROM than in PTL [1], [2], [4], [5], [9]–[14]. Although it remains unclear which first develops between substantial bacterial infection in the chorio-amniotic membranes or that in the amniotic cavity [15]–[21], ascending intrauterine infection is known to cause an inflammatory change in the chorio-amniotic membranes and sequentially elicit an inflammatory response in the amniotic cavity and fetus during the progression of acute histologic chorioamnionitis (acute-HCA). This process ultimately results in preterm parturition such as PTL or preterm-PROM [22]–[26]. Therefore, it is likely that acute-HCA, funisitis and intra-amniotic inflammation (IAI), in addition to MIAC, are more common in preterm-PROM than in PTL. Indeed, our previous studies demonstrated that acute-HCA, funisitis and IAI were more frequent in patients with preterm-PROM than in those with PTL (acute-HCA, 63% vs. 53%; funisitis, 41% vs. 25%; IAI, 42% vs. 21%), although these studies were not performed and compared simultaneously [4], [5]. However, there is a paucity of data regarding whether the intensity of a fetal and an intra-amniotic (IA) inflammatory response is more severe in preterm-PROM than in PTL, in the same context of acute-HCA with or without fetal involvement, during the progression of ascending intra-uterine infection. The purpose of this study was to examine this issue.

## Materials and Methods

### 1. Study Design

Study population consisted of 213 singleton-gestations who delivered preterm-neonates at the Seoul National University Hospital between January 1993 and December 2007 and who met the following criteria: (1) gestational age (GA) at delivery <34 weeks; (2) preterm births due to PTL (120 cases) or preterm-PROM (93 cases); (3) available results of placental histopathologic examination after delivery; (4) delivery within 4 days of amniocentesis. This criterion of amniocentesis-to-delivery interval was used to preserve a meaningful temporal relationship between the results of amniotic fluid (AF) studies, and the results of placental pathology and umbilical cord plasma C-reactive protein (CRP) concentrations obtained at birth. The intensity of fetal and IA inflammatory response was compared between patients with PTL and those with preterm-PROM according to the following placental inflammatory conditions: 1) placenta without inflammatory lesion (n = 71); 2) acute-HCA but without funisitis (n = 61); and 3) acute-HCA with funisitis (n = 81). At our institution, we routinely recommended and performed trans-abdominal amniocentesis, umbilical cord blood collection at birth, and placental histo-pathologic examination in all patients admitted with the diagnosis of PTL or preterm-PROM after written informed consent was obtained. Trans-abdominal amniocentesis was performed to evaluate either the microbiologic or inflammatory status of the amniotic cavity and/or the fetal lung maturity. The Institutional Review Board of Seoul National University Hospital approved the collection and use of these samples and information for research purposes. The Seoul National University has a Federal Wide Assurance with the Office for Human Research Protections (OHRP) of the Department of Health and Human Services of the United States. Many of patients in this study were included in our previous studies.

### 2. Clinical Characteristics of the Study Population and Diagnosis of Clinical Chorioamnionitis, Acute-HCA and Funisitis

The demographic and clinical characteristics of the mothers and their neonates were examined through a review of the medical records. We investigated maternal age, parity, GA at amniocentesis, GA at delivery, birth weight, 1 min and 5 min Apgar score as demographic and clinical characteristics.

Clinical chorioamnionitis was diagnosed when maternal temperature was elevated to 37.8°C and ≥2 of the following criteria were present: uterine tenderness, malodorous vaginal discharge, maternal leukocytosis (>15,000 cells/mm^3^), maternal tachycardia (>100 beats/min), and fetal tachycardia (>160 beats/min).

Placental tissue samples obtained for histopathologic evaluation included the chorio-amnion, the chorionic plate, and the umbilical cord. These samples were fixed in 10% neutral buffered formalin and embedded in paraffin. Sections of prepared tissue blocks were stained with hematoxylin and eosin. Pathologists were masked to the clinical information. Acute-HCA was defined as the presence of acute inflammatory changes on the examination of extra-placental membranes or chorionic plate of the placenta, and funisitis was diagnosed in the finding of the neutrophil infiltration into the umbilical vessel walls or Wharton's jelly with the use of criteria previously published [27].

### 3. The Studies of Amniotic Fluid (AF) and Umbilical Cord Plasma

AF was cultured for aerobic and anaerobic bacteria and for genital mycoplasmas (*Ureaplasma urelyticum* and *Mycoplasma hominis*) for the evaluation of MIAC according to methods previously described [28], [29]. The remaining fluid was centrifuged and stored in polypropylene tubes at −70°C. IA inflammatory response was measured by AF Matrix Metalloproteinase-8 (MMP-8) concentration. MMP-8 concentrations in stored AF were measured with a commercially available enzyme-linked immunosorbent assay (Amersham Pharmacia Biotech, Inc, Little Chalfont, Bucks, UK). The sensitivity of the test was <0.3 ng/ml. Both intra- and inter-assay coefficients of variation were <10%. Details about this assay and its performance have been previously described [30]. IAI was defined as an elevated AF MMP-8 concentration (>23 ng/mL), as previously reported [5].

Umbilical cord blood was collected in ethylene-diaminetetraacetic acid-containing blood collection tubes by venipuncture of the umbilical vein at birth. Samples were then centrifuged and supernatants were stored in polypropylene tubes at −70°C. Fetal inflammatory response was measured by umbilical cord plasma C-reactive protein (CRP) concentration at birth. Umbilical cord plasma CRP concentrations were measured with a commercially available enzyme-linked immunosorbent assay (Immunodiagnostik AG, Bensheim, Germany). The sensitivity of the test was 0.02 ng/mL. Both intra- and inter-assay coefficients of variation were <10%. Details about this assay and its performance have been previously described [31]. Fetal inflammatory response syndrome was defined as an elevated umbilical cord plasma CRP concentration (>200 ng/mL), as previously reported [31].

### 4. Statistical Analysis

Mann-Whitney U test was used for comparison of continuous variables. Comparisons of proportions were performed with the Fisher’s exact test. Statistical significance was defined as a p<0.05.

## Results

### 1. Clinical Characteristics, Pregnancy Outcomes, and the Frequency of IA Infection and Each Placental Inflammatory Condition According to PTL or Preterm-PROM

**Figure 1:**
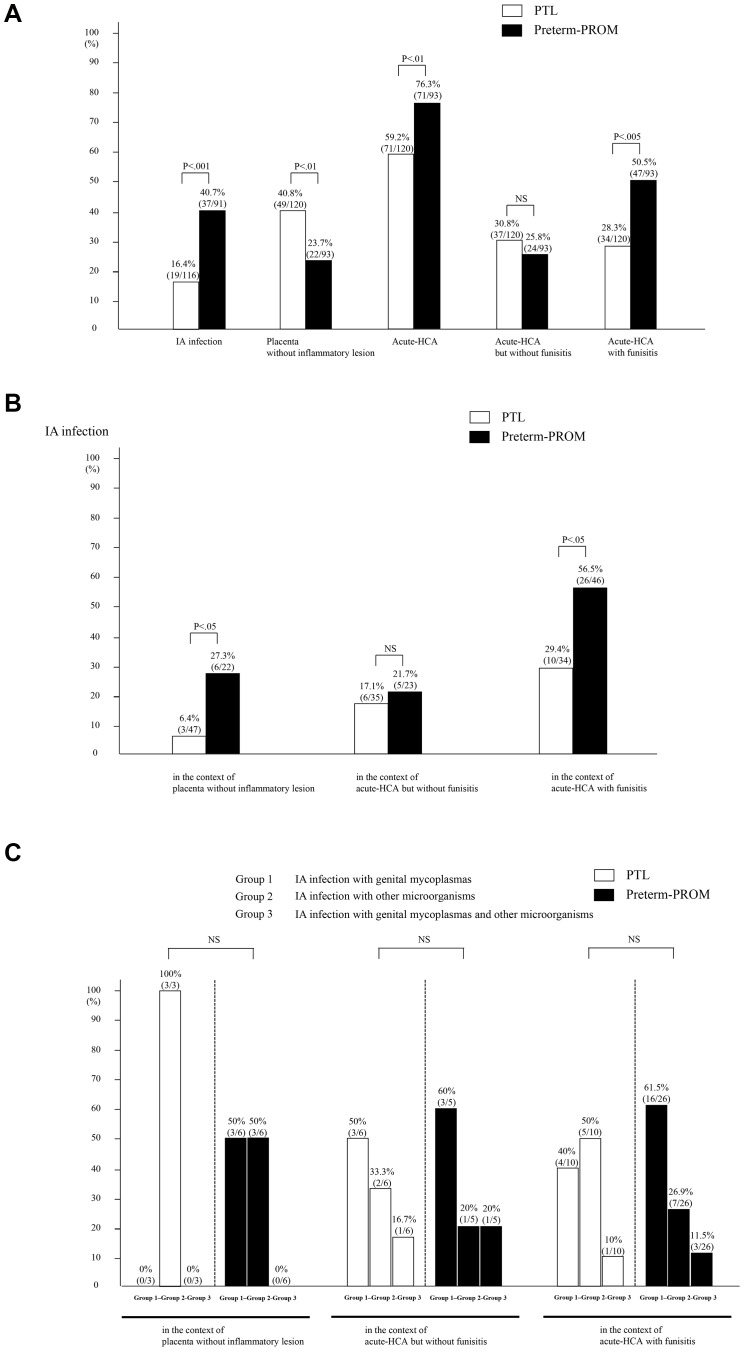
(a) The frequency of intra-amniotic (IA) infection and each placental inflammatory condition (i.e. placenta without inflammatory lesion, acute histologic chorioamnionitis (acute-HCA), acute-HCA but without funisitis, and acute-HCA with funisitis) according to preterm labor and intact membranes (PTL) or preterm premature rupture of membranes (preterm-PROM),(b) the frequency of IA infection according to PTL or preterm-PROM, in the context of the presence or absence of acute-HCA with or without funisitis (each and all frequency or P value is shown in graphs), and (c) the frequency of types of microorganisms isolated from amniotic fluid in patients with IA infection according to PTL or preterm-PROM, in the same context of placental inflammatory condition (i.e. placenta without inflammatory lesion, acute-HCA but without funisitis and acute-HCA with funisitis).

There were no significant differences in parity (≥1), maternal age, clinical chorioamnionitis, birth weight, GA at amniocentesis, 1 min Apgar score (<7) and 5 min Apgar score (<7) between patients with PTL and those with preterm-PROM (*p*>0.05 for each) (see Table 1). However, patients with preterm-PROM had a significantly higher rate of IA infection, acute-HCA and acute-HCA with funisitis (*p*<0.01 for each) (see Figure 1 (a)), and lower rate of cervical dilatation (≥2 cm, ≥3 cm and ≥4 cm) than those with PTL did (*p*<0.01 for each) (see Table 1).

**Table 1:** Clinical characteristics and pregnancy outcomes according to preterm labor and intact membranes (PTL) or preterm premature rupture of membranes (preterm-PROM).

	PTL	Preterm-PROM	*P* value
	(n = 120)	(n = 93)	
	56.3% (120/213)	43.7% (93/213)	
Mean maternal age, y (± SD)	30.4±4.4	30.0±4.0	NS
Parity ≥1	57.5% (69/120)	47.3% (44/93)	NS
Median GA at amniocentesis, wk (range)	30.0 (20.1–33.9)	31.1 (21.6–33.7)	NS
Median GA at delivery, wk (range)	30.2 (20.3–33.9)	31.4 (21.6–33.9)	<0.05
Mean birth weight, g (± SD)	1448±576	1569±592	NS
1 min Apgar score <7	66.7% (80/120)	62.0% (57/92)	NS
5 min Apgar score <7	48.3% (58/120)	34.8% (32/92)	NS
Mean Cx. dilatation at amniocentesis, cm (± SD)†	2.5±2.5	1.4±1.7	<0.0005
Cx. dilatation at amniocentesis ≥4 cm†	21.7% (25/115)	6.9% (6/87)	<0.01
Cx. dilatation at amniocentesis ≥3 cm†	42.6% (49/115)	19.5% (17/87)	<0.001
Cx. dilatation at amniocentesis ≥2 cm†	56.5% (65/115)	32.2% (28/87)	<0.001
Clinical chorioamnionitis‡	12.0% (14/117)	11.0% (10/91)	NS

*GA*, gestational age; *SD*, standard deviation; *NS*, not significant; *Cx*, cervical.

† Of 213 cases, the data about cervical dilatation at the time of amniocentesis through a review of medical records were not available in 11 cases.

‡ Of 213 cases, the data about clinical chorioamnionitis through a review of medical records were not available in 5 cases.

### 2. Clinical Characteristics, Pregnancy Outcomes, and the Frequency of IA Infection According to PTL or Preterm-PROM, in the Context of the Presence or Absence of Acute-HCA with or without Funisitis

There were no significant differences in parity (≥1), maternal age, clinical chorioamnionitis and GA at amniocentesis between patients with PTL and those with preterm-PROM, in the context of each placental inflammatory condition (placenta without inflammatory lesion, acute-HCA but without funisitis, and acute-HCA with funisitis) (*p*>0.05 for each) (see Table 2). However, IA infection was more frequent in preterm-PROM than in PTL in all placental inflammatory conditions, although it did not reach a statistical significance in cases with acute-HCA but without funisitis (see Figure 1 (b)). Moreover, it should be noted that although there were no significant differences in cervical dilatation (≥2 cm, ≥3 cm and ≥4 cm) between PTL and preterm-RPOM in the context of both placenta without inflammation and acute-HCA but without funisitis, patients with PTL had a significantly higher rate of cervical dilatation (≥2 cm, ≥3 cm and ≥4 cm) than those with preterm-PROM did in the context of acute-HCA with funisitis (p<0.05 for each) (see Table 2). Table 3 shows types of microorganisms that were isolated form AF. Ureaplasmas were the most common isolates (62.5%; 35/56). There were no significant differences in the frequency of types of microorganisms isolated from amniotic fluid between PTL and preterm-PROM among patients with IA infection in the context of placenta without inflammatory lesion, acute-HCA but without funisitis and acute-HCA with funisitis (Figure 1 (c), each for P>0.05).

**Table 2:** Clinical characteristics and pregnancy outcomes according to preterm labor and intact membranes (PTL) or preterm premature rupture of membranes (preterm-PROM) in the context of the presence or absence of acute histologic horioamnionitis (acute-HCA) with or without funisitis.

	PTL	Preterm-PROM	*P* value
Placenta without inflammation (n = 71), 33.3% (71/213)	n = 49	n = 22	
Mean maternal age, y (± SD)	30.5±4.5	29.7±3.1	NS
Parity ≥1	57.1% (28/49)	45.5% (10/22)	NS
Median GA at amniocentesis, wk (range)	31.1 (20.1–33.9)	32.2 (23.4–33.7)	NS
Median GA at delivery, wk (range)	31.1 (20.3–33.9)	32.2 (23.7–33.9)	<.05
IAI	33.3% (16/48)	38.1% (8/21)	NS
FIRS	18.6% (8/43)	9.1% (2/22)	NS
Mean Cx. dilatation at amniocentesis, cm (± SD)†	2.3±2.8	1.1±1.1	NS
Cx. dilatation at amniocentesis ≥4 cm†	20.4% (10/49)	0% (0/19)	NS
Cx. dilatation at amniocentesis ≥3 cm†	32.7% (16/49)	15.8% (3/19)	NS
Cx. dilatation at amniocentesis ≥2 cm†	49.0% (24/49)	36.8% (7/19)	NS
Clinical chorioamnionitis^a^	2.1% (1/47)	0% (0/22)	NS
Acute-HCA but without funisitis (n = 61), 28.6% (61/213)	n = 37	n = 24	
Mean maternal age, y (± SD)	29.6±3.9	29.5±3.7	NS
Parity ≥1	51.4% (19/37)	33.3% (8/24)	NS
Median GA at amniocentesis, wk (range)	28.4 (23.0–33.9)	31.1 (21.6–33.6)	NS
Median GA at delivery, wk (range)	28.6 (23.4–33.9)	31.4 (21.6–33.9)	NS
IAI	81.1% (30/37)	72.7% (16/22)	NS
FIRS	32.4% (11/34)	21.7% (5/23)	NS
Mean Cx. dilatation at amniocentesis, cm (± SD)‡	2.9±2.8	2.3±2.6	NS
Cx. dilatation at amniocentesis ≥4 cm‡	20.6% (7/34)	18.2% (4/22)	NS
Cx. dilatation at amniocentesis ≥3 cm‡	47.1% (16/34)	31.8% (7/22)	NS
Cx. dilatation at amniocentesis ≥2 cm‡	61.8% (21/34)	50.0% (11/22)	NS
Clinical chorioamnionitis^b^	13.9% (5/36)	4.5% (1/22)	NS
Acute-HCA with funisitis, (n = 81), 38.0% (81/213)	n = 34	n = 47	
Mean maternal age, y (± SD)	31.1±4.6	30.4±4.4	NS
Parity ≥1	64.7% (22/34)	55.3% (26/47)	NS
Median GA at amniocentesis, wk (range)	29.4 (23.6–33.4)	30.7 (21.6–33.7)	NS
Median GA at delivery, wk (range)	29.5 (23.9–33.6)	30.9 (21.9–33.9)	NS
IAI	100% (31/31)	88.4% (38/43)	NS
FIRS	74.2% (23/31)	61.9% (26/42)	NS
Mean Cx. dilatation at amniocentesis, cm (± SD)∮	2.3±1.7	1.1±1.2	<0.005
Cx. dilatation at amniocentesis ≥4 cm∮	25.0% (8/32)	4.3% (2/46)	<0.05
Cx. dilatation at amniocentesis ≥3 cm∮	53.1% (17/32)	15.2% (7/46)	<0.0005
Cx. dilatation at amniocentesis ≥2 cm∮	62.5% (20/32)	21.7% (10/46)	<0.0005
Clinical chorioamnionitis	23.5% (8/34)	19.1% (9/47)	NS

*GA*, gestational age; *SD*, standard deviation; *NS*, not significant; *IAI*, intra-amniotic inflammation; *FIRS*, fetal inflammatory response syndrome; *Cx*, cervical.

† Of 71 cases, the data about cervical dilatation at the time of amniocentesis through a review of medical records were not available in 3 cases.

‡ Of 61 cases, the data about cervical dilatation at the time of amniocentesis through a review of medical records were not available in 5 cases.

∮ Of 81 cases, the data about cervical dilatation at the time of amniocentesis through a review of medical records were not available in 3 cases.

a Of 71 cases, the data about clinical chorioamnionitis through a review of medical records were not available in 2 cases.

b Of 61 cases, the data about clinical chorioamnionitis through a review of medical records were not available in 3 cases.

**Table 3:** Types of micro-organisms isolated from amniotic fluid in patients with intra-amniotic infection among study population.

Microorganism	Group-Cases, n	Cases, n
Group 1 (intra-amniotic infection with genital mycoplasmas)	29	
Ureaplasmas		28
Ureaplasmas and *M hominis*		1
Group 2 (intra-amniotic infection with other microorganisms)	21	
* Candida* species		5
* Staphylococcus aureus*		2
* Streptococcus anginosus* group		2
* Klebsiella pneumoniae*		2
* Corynebacterium* species		2
* Lactobacillus* species		2
* Burkholderia cepacia* complex		1
Group B *Streptococcus*		1
* E coli*		1
* Viridans Streptococcus and Corynebacterium* species		1
* Acinetobacter* species		1
* Gardnerella vaginallis*		1
Group 3 (intra-amniotic infection with genital mycoplasmas and other microorganisms)	6	

### 3. AF MMP-8 Concentrations and Umbilical Cord Plasma CRP Concentrations at Birth According to PTL or Preterm-PROM, in the Context of the Presence or Absence of Acute-HCA with or without Funisitis

**Figure 2:**
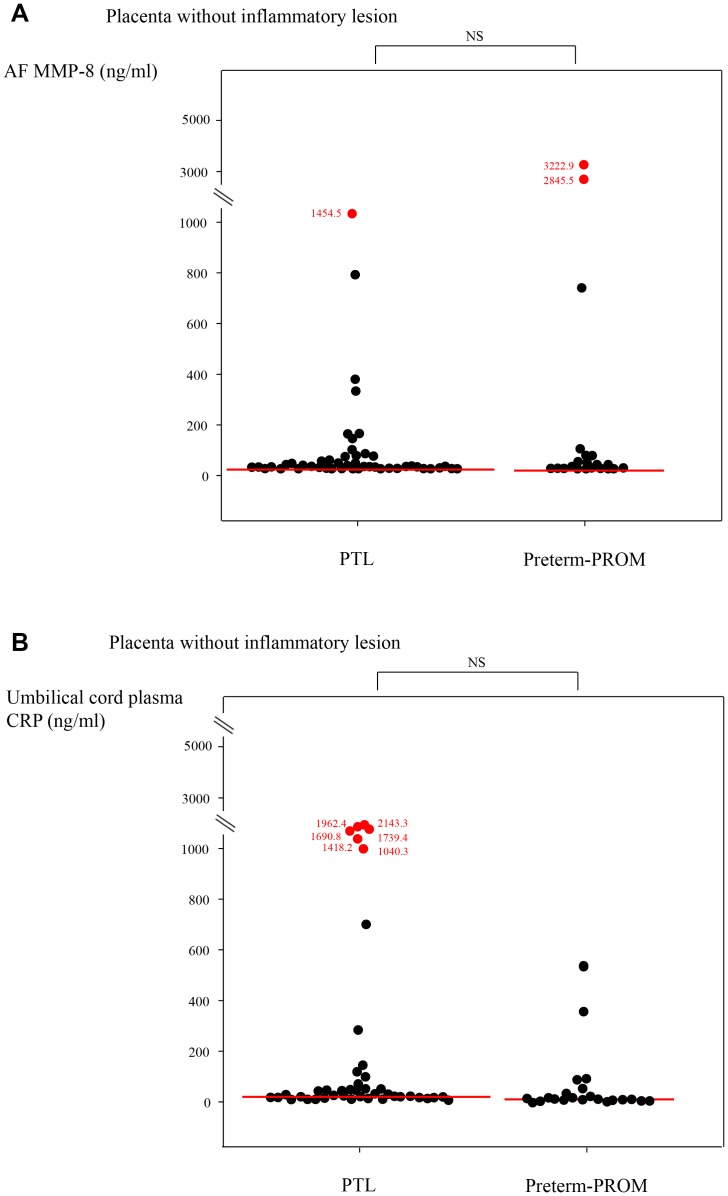
(a) AF MMP-8 concentrations and (b) umbilical cord plasma CRP concentrations at birth according to PTL or preterm-PROM in the context of placenta without inflammatory lesion (AF MMP-8: PTL, median, 9.5 ng/ml [range, 0.3–1454.5 ng/ml] vs. preterm-PROM, median, 9.1 ng/ml [range, 0.3–3222.9 ng/ml]; umbilical cord plasma CRP: PTL, median, 21.9 ng/ml [range, 0.2–2143.3 ng/ml] vs. preterm-PROM, median, 14.7 ng/ml [range, 0.3–522.7 ng/ml]; each for p = NS).

**Figure 3:**
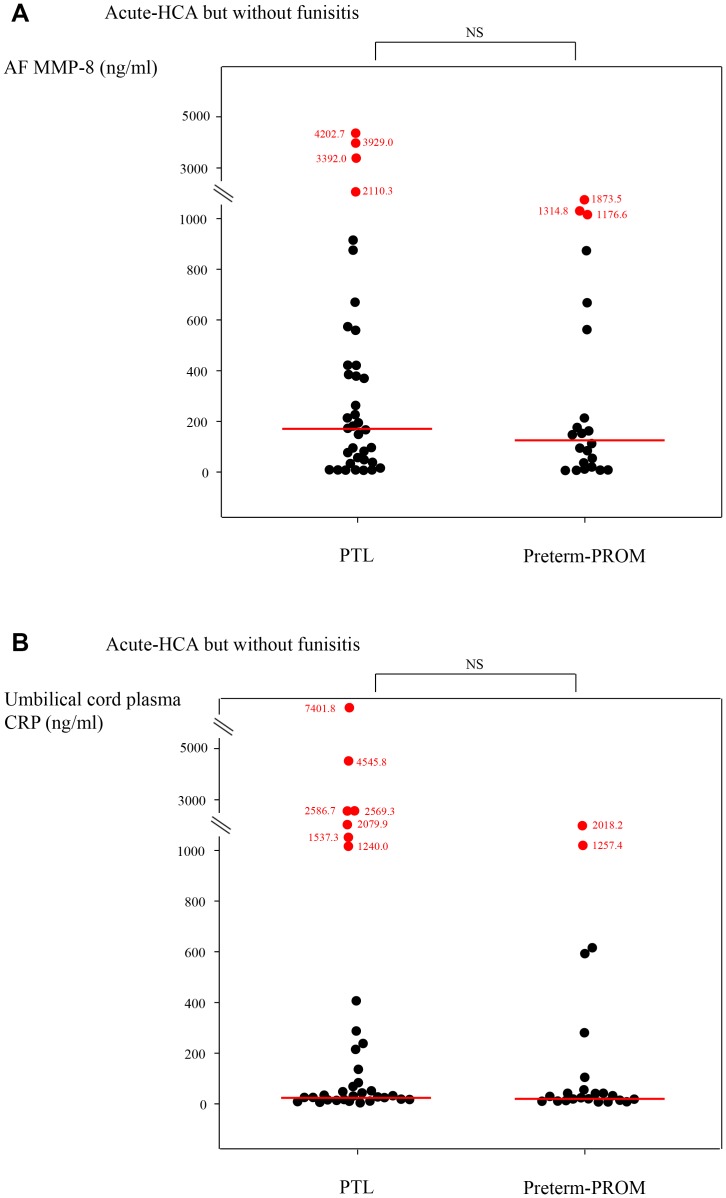
(a) AF MMP-8 concentrations and (b) umbilical cord plasma CRP concentrations at birth according to PTL or preterm-PROM in the context of acute- HCA but without funisitis (AF MMP-8: PTL, median, 175.4 ng/ml [range, 1.1–4202.7 ng/ml] vs. preterm-PROM, median, 124.0 ng/ml [range, 0.3–1873.5 ng/ml]; umbilical cord plasma CRP: PTL, median, 37.0 ng/ml [range, 2.9–7401.8 ng/ml] vs. preterm-PROM, median, 27.9 ng/ml [range, 5.6–2018.2 ng/ml]; each for p = NS).

**Figure 4:**
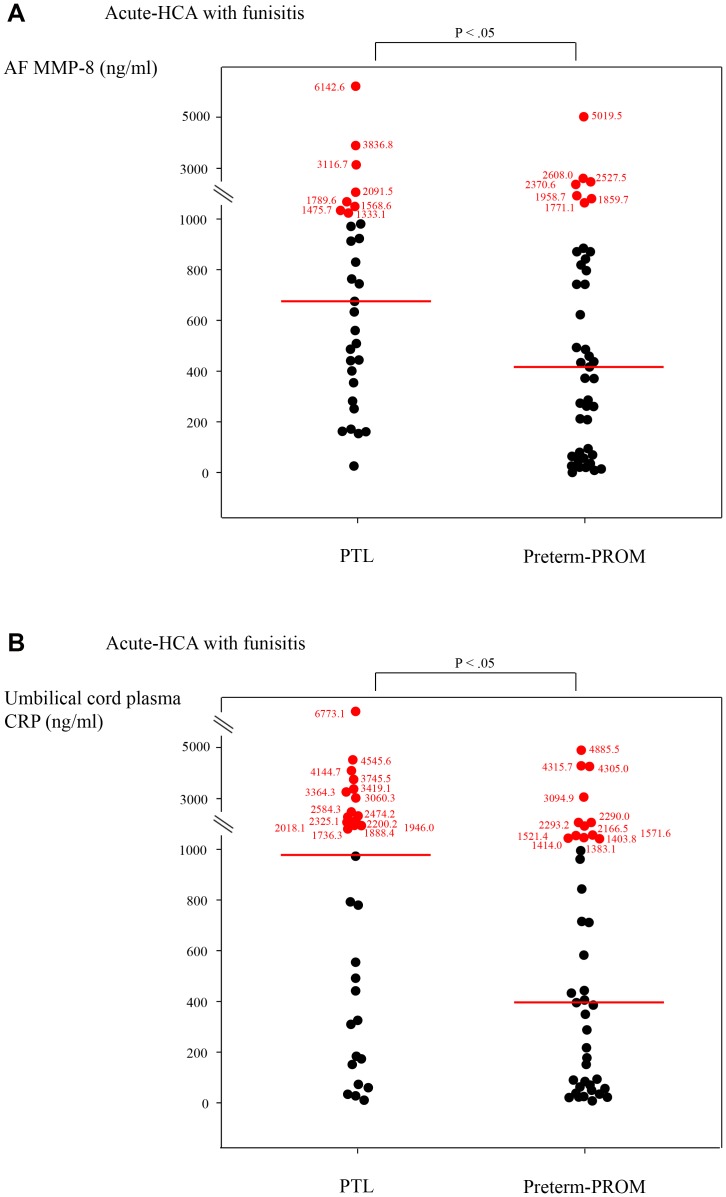
(a) AF MMP-8 concentrations and (b) umbilical cord plasma CRP concentrations at birth according to PTL or preterm-PROM in the context of acute- HCA with funisitis (AF MMP-8: PTL, median, 675.0 ng/ml [range, 26.0–6142.6 ng/ml] vs. preterm-PROM, median, 416.8 ng/ml [range, 0.4–5019.5 ng/ml] (p<.05); umbilical cord plasma CRP: PTL, median, 969.3 ng/ml [range, 7.6–6773.1 ng/ml] vs. preterm-PROM, median, 396.9 ng/ml [range, 4.9–4885.5 ng/ml]; each for p<.05)

There were no significant differences in a median AF MMP-8 concentration and umbilical cord plasma CRP concentration at birth between patients with PTL and those with preterm-PROM, in the context of both placenta without inflammatory lesion (see Figure 2) and acute-HCA but without funisitis (see Figure 3) (for each p>.05). However, PTL was associated with a higher median AF MMP-8 concentration and umbilical cord plasma CRP concentration at birth than preterm-PROM, in the context of acute-HCA with funisitis (AF MMP-8∶675.0 ng/ml [26.0–6142.6 ng/ml] vs. 416.8 ng/ml [0.4–5019.5 ng/ml]; umbilical cord plasma CRP: 969.3 ng/ml [7.6–6773.1 ng/ml] vs. 396.9 ng/ml [4.9–4885.5 ng/ml]; for each p<.05) (see Figure 4).

## Discussion

The principal findings of this study are as following. Firstly, patients with preterm-PROM had higher rates of IA infection, acute-HCA, and acute-HCA with funisitis than those with PTL did. Secondly, there were no significant differences in the fetal and IA inflammatory responses and the cervical dilatation between patients with PTL and those with preterm-PROM in the context of both placenta without inflammatory-lesion and acute-HCA but without funisitis. Thirdly, acute-HCA with funisitis was associated with a significantly higher fetal and IA inflammatory response and increase in cervical dilatation in PTL than in preterm-PROM, despite less common IA infection in PTL than in preterm-PROM.

Our previous studies reported that a fetal inflammatory response was stronger in patients with MIAC than in those without MIAC among cases with both preterm-PROM and IAI [32], and an IA inflammatory response was more intense in AF with genital mycoplasmas than in AF with other microorganisms among cases with both preterm-PROM and IA infection [33]. However, there is a paucity of data regarding whether the impact of MIAC on a fetal and an IA inflammatory response is similar or different between PTL and preterm-PROM. This study shows that there were no significant differences in fetal and IA inflammatory responses between PTL and preterm-PROM, in the context of placenta without inflammatory-lesion, despite more common IA infection in preterm-PROM than in PTL. Moreover, our data paradoxically demonstrated that a fetal and an IA inflammatory response was more severe in PTL than in preterm-PROM in the context of acute-HCA with funisitis, despite less common IA infection in PTL than in preterm-PROM. Possible explanations for our unexpected findings are as following. Firstly, in addition to placental inflammatory condition, an increase in cervical dilatation, may be accompanied by the intense fetal and IA inflammatory response. Indeed, patients with PTL had a significantly increase in cervical dilatation than those with preterm-PROM did in the context of acute-HCA with funisitis. Moreover, it should be noted that there was no significant difference in cervical dilatation between PTL and preterm-PROM in the context of both placenta without inflammatory lesion and acute-HCA but without funisitis, which did not cause the differences in the intensity of fetal and IA inflammatory responses between PTL and preterm-PROM. This interpretation parallels the previous findings that the risk of IAI increases after the cervix begins to dilate in term pregnant women with regular uterine contractions with intact membranes [34], and that the more advanced the cervical dilatation, the greater the risk of a higher median AF white blood cell (WBC) count in women at term with intact membranes [35]. Secondly, the pathogenesis of PTL and preterm-PROM may be fundamentally different. The difference in inflammatory mediators between PTL and preterm-PROM may precede the onset of labor and determine who would and would not go into labor. Moreover, preterm-PROM may be more common in the context of specific microorganisms that may incite less cytokine production into the AF, but ultimately weaken the membranes sufficiently to cause rupture.

Major strengths of this study are: (1) its large cohort (n = 213) of consecutive singleton preterm gestations (<34 weeks) due to PTL or preterm-PROM; (2) it included several markers of infection and an inflammatory response, including AF culture, AF MMP-8 concentration, umbilical cord plasma CRP concentration at birth and acute-HCA with or without funisitis, and therefore, this study analyzed the inflammatory responses in the relevant potential compartments among study groups; and (3) it compared a fetal and an IA inflammatory response between PTL and preterm-PROM, in the same context of various aspects as followings: (1) delivery within 4 days of amniocentesis; (2) the extent of placental inflammatory conditions (i.e., placenta without inflammatory lesion, acute-HCA but without funisitis, and acute-HCA with funisitis); (3) others: maternal age, parity (≥1) and GA at amniocentesis. Therefore, patients with PTL and those with preterm-PROM can be comparable groups for analyzing a fetal and an IA inflammatory response by virtue of obstetric management. The potential weaknesses of the study are: (1) it did not analyze neonatal morbidity according to PTL or preterm-PROM, in the context of each placental inflammatory condition, due to the following reasons: the relatively small sample size for the evaluation of neonatal morbidity and extending beyond the scope of this study; and (2) it did not perform the broad range PCR or more sophisticated techniques of microbial identification in addition to culture technique. Culture is fairly insensitive and mistakes in organism identification are not uncommon. Therefore, the results on the differences in microorganisms between PTL and preterm-PROM in current study are thought to be extremely preliminary.

Our data demonstrated the novel finding that PTL was associated with greater fetal and.

IA inflammatory responses than preterm-PROM, in the context of acute-HCA with fetal involvement, despite less common IA infection in PTL than in preterm-PROM. This finding has clinical and patho-physiologic implications in that the differences in the intensity of a fetal and an IA inflammatory response between PTL and preterm-PROM may suggest the fundamental difference in the pathogenesis between the two conditions, although the exact mechanisms responsible for this unexpected clinical observation should be investigated.

The differentiation between PTL and preterm-PROM should be made in the research about the patho-physiology of preterm births as well as in the management of patients at risk for preterm births. Moreover, it should be determined whether the mere PTL itself may increase a more IA inflammatory response than preterm-PROM, or whether much higher intensity of AF MMP-8 concentration may act preferentially on the production of prostaglandin for PTL, rather than on the degradation of extracellular matrix in the chorio-amniotic membranes for preterm-PROM. However, it is difficult to perform these studies in human population, and therefore, in vitro or animal experiments regarding above unexplained issues may be required.
